# Feasibility Analysis of Bolted Joints with Composite Fibre-Reinforced Thermoplastics

**DOI:** 10.3390/polym13121904

**Published:** 2021-06-08

**Authors:** Daniel Tobalina-Baldeon, Felix Sanz-Adán, Marian Martinez-Calvo, Carmelo Gómez, Inigo Sanz-Pena, Francisco Cavas

**Affiliations:** 1 Department of Mechanical Engineering, University of La Rioja, 26004 Logroño, Spain; Daniel.tobalina@alum.unirioja.es (D.T.-B.); marian.martinez@unirioja.es (M.M.-C.); 2 International School of Doctorate, Technical University of Cartagena, 30202 Cartagena, Spain; gomezgarciacarmelo@gmail.com; 3 Department of Mechanical Engineering, Imperial College London, London SW7 2AZ, UK; i.sanz-pena@imperial.ac.uk; 4 Department of Structures, Construction and Graphical Expression, Technical University of Cartagena, 30202 Cartagena, Spain; francisco.cavas@upct.es

**Keywords:** polymer characterization, CFRTP, polymeric structures, bolted joint, top-mount, gear-mount, stiffness loss

## Abstract

The use of composite materials has shown steady growth in recent years due to their excellent specific mechanical properties and the possibility to reduce the weight of vehicles without impairing their safety and comfort. Continuous fibre-reinforced thermoplastic composites (CFRTP) show dynamic, acoustic, and damping properties far superior to steel and can be recycled and repaired. Their excellent properties make CFRTP good candidates for anti-vibration and shock absorbing components, however, out-of-plane mechanical properties hinder the anchoring to the vehicle’s body by means of bolted connections. The results obtained in this study show how the maximum torque that can be applied without cracks or breakage phenomena is lower than in standard steel joints. Although the preload’s value is admissible, this one is reduced over time due to relaxation phenomena associated with the viscoelastic behaviour of thermoplastic matrix. The results obtained can be improved with the integration of metal inserts in connections’ areas. In this study, a case study of a gear mount replacing the steel core with CFRTP reinforced with inserts is carried out. The results show a reduction above 50% in weight, opening the possibility of lighter structures in the automotive sector.

## 1. Introduction

As sustainability has become a key factor in the automotive sector [1], in recent years, considerable efforts have been made to reduce weight by increasing, at the same time, the number of functionalities. Accordingly, composite applications have steadily been incremented given their excellent specific properties, and the possibility of being recycled, joined and repaired. Kuram, E. et al. [2] showed that a binary blend of Polybutylene terephthalate/polycarbonate reinforced with short glass fibre can be re-injected in a recycled process without loss of mechanical properties. Furthermore, the use of thermoplastic composites opens the possibility to be joined and repaired in volume-intensive applications, a key factor in the automotive sector, where short processing cycles are required [3].

Components made of thermoplastics materials that cannot be repaired or reused can be shredded and used in other sectors. Şimşek, B. and Uygunoğlu, T. [4] studied the use of polymers in concrete applications. The results obtained in this study show how the addition of polycarbonate in concrete allows one to improve thermal isolation and reduce water absorption, useful in applications with water resistance requirements.

Composite materials are used in the automotive sector in non-structural elements such as dashboards, rear-view mirrors, and in the body of high-end vehicles customized for small series production.

The massive incorporation of electronic systems in vehicles has allowed the inclusion of thermoplastic materials thanks to their excellent dielectric and electric insulation properties. Thermoplastic materials can be mass-produced and supplied on rigid sheets for easy distribution, storage and processability, improving logistics and production capacity. Thermoplastic material properties can be modified by blending with other polymers and additives to improve their behaviour against fire. Roenner, N. et al. [5] showed that the use of inert additives like hollow glass spheres and boron nitride platelets can improve polymers flammability characteristics whereas Yang, W. et al. [6] showed how the substitution of a fraction of aluminium hypophosphite (AHP) by Nanoclay can be useful to obtain enhanced fibre-retarded performance in glass-fibre-reinforced polybutylene terephthalate components in a more eco-friendly way. Furthermore, the blend with other polymers can improve the impact behaviour. Zhang, D. et al. [7] analysed the influence of thermoplastic polyurethane (TPU) in long glass fibre-PBT components. The incorporation of TPU allowed an increment in tensile and notched Izod impact strength with a small reduction in the flexural mechanical properties.

Composite fibre-reinforced thermoplastics (CFRTP) exhibit good mechanical properties and are, thus, a promising alternative in the automotive sector to replace metallic components.

**Figure 1:**
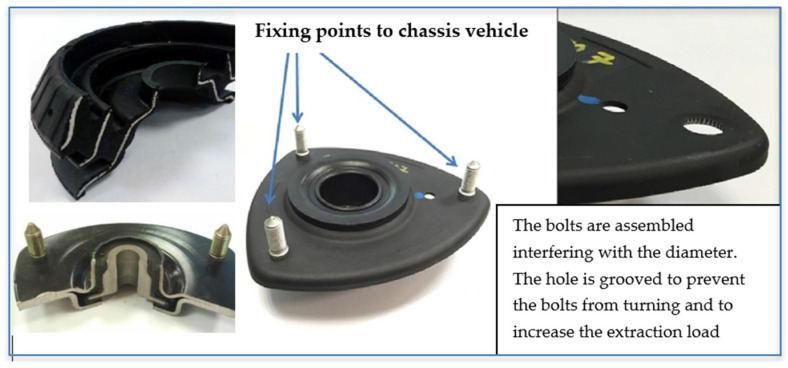
Anti-vibration and damping components with steel plates.

The improved behaviour of CFRTP components leads to better comfort performance by reducing vibrations and noise when used in structural elements with high stiffness requirements, such as connecting rods to top mounts. Tobalina-Baldeon et al. [8] analysed the dynamic tensile-compressive stress behaviour of PA6 reinforced with 45% (in volume) of continuous fibreglass and PA66 reinforced with 45% (in volume) of continuous carbon-fibre in structural car elements with antivibration and damping functionalities (Figure 1). Their results showed that CFRTP components offer dynamic advantages over the materials currently used as antivibration elements. Furthermore, impact behaviour can be improved by incorporating self-healing elements. Williams et al. [9] investigated the effect of embedded hollow glass fibres (HGF) in laminates under quasi-static impacts. Their results showed that HGF incorporation increases energy absorption and, hence, the degree of damage tolerance.

The structural efficiency of composite structures depends mostly on the joints. Typical methods involve employing rivets, bolts, adhesives, and clinching and welding in CFRTP metal joints. Many research studies have been conducted about the obtained methods, processes, mechanisms and properties of CFRP composite and aluminium alloys [10].

Most of the common joints between metal–composite structures are usually based on bonded and bolted connections, where the load is transferred to the laminate plane. Since the classic analysis by O. Volkersen [11] where rivet-force distribution in tension-stressed riveted joints was analysed, considerable efforts have been made to improve the strength of composite joints. L.J. Hart Smith [12] analysed bonded and bolted joints to demonstrate that an adhesive bond load path is much stiffer, and the combination of bolted and bonded joints is no stronger than a well-designed bonded joint alone. However, standard bonded joints based on adhesive applications present a limited thickness to be joined and are prone to environmental degradation. Furthermore, this kind of joint cannot be disassembled and require specific quality control.

Bonded strength depends on adherent and adhesive strengths, and the bonded configuration adopted to reduce peeling stress improves the fatigue behaviour. Ishii et al. [13] investigated adhesively bonded CFRP-metal joints and showed that fatigue limits are determined by the maximum principal stress. Tobalina-Baldeon et al. [14] proposed a methodology for testing and characterising a bonding joint between rigid substrates made of CFRTP and over-injected vulcanized rubber.

Strength in bonded joints can be improved with pre-treatments over metallic materials. Laser applications to metal surfaces allow the creation of microstructures with undercut grooves [15]. Zhang et al. [16] showed how aluminium pre-treatment improved the shear strength of aluminum-PA6 sheets by about four-fold increase. Scarselli et al. [17] found that applying plasma and UV treatments to PEEK and PPS joints improves the strength of the bond.

The use of composites based on a thermoplastic matrix allows joints to be welded in this way. Several research studies have been published about laser and friction welding procedures. Bolt S. [18] carried out an experimental study to examine the application of ultrasonic plastic welding in hybrid joints between metals and carbon fibre-reinforced polyamide 6. Test results proved an average single lap shear strength of 8 MPa. Suzuki, K., et al. [19] performed a numerical study of thermoplastics’ joints made with ultrasonics techniques. The behaviour of metal–plastic bonded joints obtained by laser heating depends on the applied energy density [20], and the obtained porosity type [21]. Air bubbles around the joints may be caused by resin matrix thermal decomposition [22]. Besides laser applications, welding connections between CFRTP-metals can be achieved by means of friction. Lambiase et al. [23] investigated the influence of clamping frame material on the friction-assisted joining of Al-Mg aluminium plates with polyvinyl-chloride (PVC) sheets. They showed how using a clamping frame made of wood enables a faster heating process and a uniform process to increase the obtained joint’s mechanical strength.

Although laser and frictional welding procedures can be a useful set of techniques for bonding CFRTP-metals components, the need for assembled and inspection procedures has led to bolted connections being applied, where many studies have been carried out in aerospace applications. McCarthy, C.T. and McCarthy, M.A. [24] carried out experimental and numerical analysis of bolted connections. The results obtained show the influence of bolt–hole clearance in the stress distribution obtained for multi-bolt joints. Ramkumar, R., et al. [25] developed an analytical design methodology taking into account several joint parameters. Typically, bolted joints with in-plane load transfer, composites are locally reinforced to achieve a quasi-isotropic configuration to improve bearing strength. Furthermore, both the distance between bolts and the relationship between the joint thickness and the bolt’s diameter have been analysed in detail [26]. In standard applications, bolted connections are adequately adapted to avoid fatigue failure, fretting and corrosion of metal components, which must be properly adapted depending on the materials they come into contact with.

Bolted joints have been analysed according to published methodologies based on bolted single lap-type specimens [27]. In these analyses, shear loads are applied until tearing occurs. In this way, studies are often extrapolated from tests according to the ASTM Standard for bolted joints between metals [28]. For composite materials, material failure theories are usually based on phenomenological models. Tsai and Wu proposed quadratic failure criteria [29] to analyse the strength of composite laminates, whereas Puck and Schürmann [30] proposed criteria to differentiate fibre and inter-fibre failure in order to evaluate the fracture angle. DeTeresa et al. [31] analysed the moderate compression level effects on the behaviour of the interlaminar strength of composite structures, which improved. Tobalina et al. [32] proposed a test method to determine the adhesion values of composite/rubber material. They demonstrated that thermoplastic matrix PA66 reinforced with carbon fibre material shows an interlaminated failure mode, which occurs earlier than with the fibreglass material PA6.

However, CFRTP-metals joints are reliant on the polymers’ durability, whose mechanical properties are prone to environmental degradation and worsen with time and temperature, as it was described by Gómez et al. [33]. This study demonstrated that the addition of glass fibres makes it possible to reduce the loss of stiffness over time and increase the energy dissipated in each deformation. Furthermore, out-of-plane mechanical properties and material relaxation mechanisms due to the viscoelastic behaviour of thermoplastic matrix, hinder CFRTP components from being anchored by bolted connections. The mechanical properties of CFRTP materials are still lacking, and the limited data provided by manufacturers are not always validated.

The thermoplastic matrix PA66 reinforced with carbon fibre material shows, in most of the cases, an interlaminate failure mode, and in all of the situations happens before or in a higher percentage than with the fibreglass PA6 material.

Keeping the same combination of composite/adhesive but changing the type of rubber, which involves a lower tensile strength, the maximum breakage load does not change but the displacements increase and the failure mode is completely different. Based on the results, it concluded that the third test method is appropriate to determinate the adhesion values of the composite material/rubber joint. However, it seems that the maximum load will never exceed 11 kN in both composite materials since the load is causing an interlaminate failure.

The review of the literature reflects the importance of threaded joints in the implementation of thermoplastic polymers in vehicles, such as damping and antivibration structural elements. To implement thermoplastic polymers, it is necessary to understand the material behaviour throughout its life cycle. This study presents a fatigue analysis of the CFRTP/Steel-threaded joint from a vehicle chassis application based on the results obtained in previous studies from the authors in anti-vibration and damping [8], CFRTP-rubber adhesive [14], viscoplasticity and high-temperature stiffness [33], and characterization of a composite material reinforced with vulcanized rubber [32].

## 2. Materials and Methods

### 2.1. General Features

The maximum torque that can be applied in bolted joints and the loss of torque allowance due to fatigue from cyclic traction–compression conditions are key factors for the application of CFRTP in antivibration and shock absorber components. Carlsson, Adams et al. [34] described in Chapter 5 the preparation of test specimens and explained the testing equipment required for mechanical testing.

In this study, the behaviour of CFRTP components, after being subjected to a fixed torque for a long period of time, were analysed and compared with steel components under the same conditions to verify if the maximum torques reached produced cracks or the breakage of the composite component.

The tests carried out were performed following the standards defined by an automotive manufacturer (The Volkswagen Group, Wolfsburg, Germany) [35,36] as references to determine the tightening torque based on the characteristics of both the thread and bolted joint package (Table 1).

The experimental protocol is described as follows:

1. Selection of the Composite material for the antivibration and damping structural elements application. The material selection criteria are based on their mechanical properties and dynamic advantages [37].
2. Implementation of experimental tests to measure the maximum torque in different CFRTPs (The methods and results can be found in Section 2.2 and Section 3.1 of this study).
3. Implementation of experimental tests to evaluate the loss of tightening (The methods and results can be found in Section 2.3 and 3.2 of this study).
4. Implementation of durability tests based on cyclic loads in a CFRTP automotive component with the bolted joint application (Section 3.3).
5. Implementation of durability test for hybrid metal-CFRTPs solutions to substitute steel parts in antivibration components and shock absorbers in automobiles using bolted joints (Section 3.3).

### 2.2. Maximum Torque Test

Maximum torque tests were carried out with a dry threaded joint based on bolts with a nominal diameter of 12 mm (M12) and 12.9 strength grade. Thus, high torques can be applied ensuring that the composite breakage occurs before the bolt breakage, and allowing maximum torque values higher than in automotive bolted joints applications, which normally use bolts with a nominal diameter of 10 mm (M10) and 10.9 strength grade. The thickness of the steel plates used in the top mount varies between 2 and 3 mm. In this study, composite specimens with a thickness of 2.5 mm and 3 mm were tested under the most critical conditions to assess the maximum safety criteria.

The tests were carried out in four different materials:

- PA66 FG30 Polyamide Reinforced with 30% random short fibreglass.
- CFRTP PA6 FG45 Polyamide Reinforced with 45% continuous fibreglass.
- CFRTP PA66 CF45 Polyamide Reinforced with 45% continuous carbon fibre.
- C45 quenched and tempered steel plate (according to ASTM-1045 and EN10083/2), which composes the structure material part to which the FRTP component is anchored.

**Figure 2:**
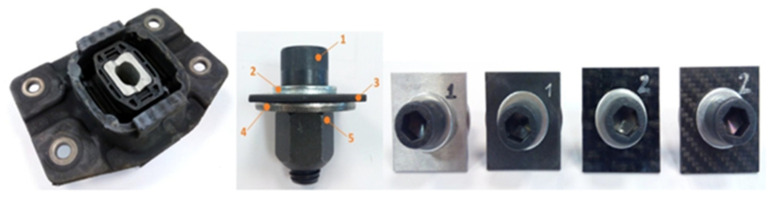
The torque test specimen assembly following a dry mounting configuration (bolt, nut, washers, metal part, and composite part).

The tightening torque applied to the specimens is based on the methodology defined in the automotive sector for testing steel specimens. This method was followed considering that no previous data or established standards for CFRTPs bolted joints have been previously defined. The joining elements and the specimens of the different materials employed in the tests are shown in Figure 2.

The tightening torque tests were carried out with and without the use of a washer in the joint. Thus, we analysed the influence of this element on both metal–composite interface and the final mechanical joint deformation. The components’ assembly is shown in Figure 2 where all the attached fastening elements and different tested materials are represented.

After assembling all the parts, the tightening torque was applied using a torque wrench with a double-needle torque meter until the tested specimen breakage occurred.

The components of the specimen assembly shown in Figure 2 are the following:

1. M12 Allen bolt with cylindrical head and strength grade 12.9 (ISO 4762).
2. Washer (Ø 24 mm × Ø 13 mm × 2.5 mm).
3. The CFRTP specimen that models the product plate (40 mm × 35 mm × 2 mm).
4. C45 steel circular plate that models the structural steel part of the vehicle at the joint. For M12, it is Ø 24 mm × Ø 12.2 mm × thickness 2.5 mm.
5. M12 nut of class 12.9.

### 2.3. Loss of Torque Test

The tightening torque is usually applied by means of a torque wrench with dynamic torque measurement. The applied torque is usually set based on standards given by the product’s manufacturer. However, the reached value does not account for the tension caused by pulling the bolt and the compression of the steel and composite plates, which was experimentally found to be approximately 10% of the applied force in steel joints (0.1 × F_M_), and defines the preload force. However, this method does not take into consideration the application and characteristics of the bolted joint.

The experimental characterization of composite materials has made big steps over recent years. Carlsson et al. [34] described different test specimen preparations, measurement devices and testing machines, obtaining accurate experimental results. An excessive preload can produce transverse crack initiation depending on the laminate configuration as was shown in a study on the fatigue life prediction in CFRP [38].

The theoretical maximum tightening torque that can be applied to the tested specimens was calculated considering the thread characteristics and two theoretical methods. Furthermore, the obtained theoretical maximum torque was compared and verified with Standards EN and ISO.

The maximum torque values obtained for M12 bolts with a strength grade of 12.9 and M10 bolts with a strength grade of 10.9 are shown in Table 1, along with the values used by an automotive OEM (original equipment manufacturer) [35,36] and the values recommended for isometric threaded joints [39].

Technical literature indicates an early method with a simplified formula (Equation (1) to obtain the tightening torque (M*_A_*). This methodology can only be applied during the elastic behaviour of the material. The relationship between the tightening torque and the preload force generated by the bolt (F*_M_*) is calculated taking into account the nominal diameter (d) and an empirical constant called the nut factor (K is a dimensionless quantity), which varies depending on the friction coefficient of the bolt with the joint (steel- and/or zinc-plated threads = 0.2; lubricated threads = 0.16).

(1) $$M_{A}=K\times d\times F_{M}$$

The maximum applied preload value in Equation (1) was determined from the value of the allowable tensile stress of the bolt determined by Equation (2):

(2) $$F_{M}=R_{p0.2}\times \frac{\pi }{4}\times {\left(\frac{d_{2}+d_{3}}{2}\right)}^{2}≅0.9\times R_{m}\times \frac{\pi }{4}\times {\left(d-0.938194\times p\right)}^{2}$$

where:

- F_M_ = Force applied to the head of the screw or the nut [N].
- R_m_ = Tensile strength [N].
- R_p0.2_ = Stress at 0.2% non-proportional elongation yield; 90% Rm (ISO 898 [40]).
- d_2_ = pitch diameter of the thread [mm] (ISO 724).
- d_3_ = core diameter of the thread.
- d_2_ and d_3_ according to the nominal diameter (*d*) and thread pitch (*p*).

According to the standard VDI 2230 [37], the assembly preload and the tightening torque can be obtained with a more accurate method taking into account the bolt and hole geometry, the material, and the thread–thread and head–seat friction coefficients. The tightening torque (M_A_) is the sum of the friction torques at the thread and the bolt head (Equation (3)).

(3) $$M_{A}=M_{G}+M_{K}$$

M_G_ is the thread torque and M_K_ is the friction torque at the screw head/nut interface [N·m]. The relationship between the tightening torque and the preload assembly is defined by Equation (4).

(4) $$M_{A}=F_{M}\times \left(\frac{P}{2\cdot \pi }+0.5775\times d_{2}\times \mu_{G}+\frac{D_{\mathrm{KM}}}{2}\times \mu_{K}\right)$$

(5) $$F_{M}=\frac{0.9\times \frac{\pi }{4}\times d_{s}^{2}}{\sqrt{1+3\times [1.5\times \frac{d_{2}}{d_{s}}\cdot {\left(\frac{P}{\pi \times d_{S}}+1.155\times \mu_{G}\right)]}^{2}}}$$

where:

- M_A_ = Moment/torque applied to the bolt.
- F_M_ = Preload force on the thread.
- D_KM_ = Average friction diameter of the annular sliding area of the head or nut (for standard M12 = 15.5 mm).
- d_s_ = Strength thread diameter (10.3602 mm for M12).
- d_2_ = Thread pitch diameter (d2 = d − 0.6495p, 10.86 for M12).
- p = Thread pitch (1.75 for M12).
- µ_G_ = Dry thread–thread friction coefficient (approx. the equal to µ*_K_*).
- µ*_K_* = Head or nut friction coefficient against the base (dry = 0.12 to 0.20, where 0.12 is considered the most unfavourable).

The total stress on the bolt is caused by the preload force and the torsional stresses, which determine the maximum tightening torque allowance.

The nut factor K used in Equation (1) and obtained with the general tables provided by manufacturers is related with the expression in the brackets of Equation (4) by means of the nominal diameter (d) of the thread according to Equation (6).

(6) $$K=\frac{\left(\frac{p}{2\pi }+0.5775\times d_{2}\times \mu_{G}+\frac{D_{\mathrm{KM}}}{2}\times \mu_{K}\right)}{d}$$

Additionally to the two methods previously mentioned, the maximum torque value was calculated according to the Standard VW 01126-1 [35]. The equations defined in this standard refer to the values below the yield strength in the elastic behaviour region. The standard previously mentioned provides a table with maximum torque values based on the metric used for components’ assembly. The obtained values were contrasted with those of Standard VW 01126-2 [36], which provides the values of tightening torques for the threaded assemblies beyond the yield point.

The tightening torques and the assembly preloads are shown in Figure 1 for the joints using M12 (strength grade of 12.9) and M10 (strength grade of 10.9) bolts. The differences in the maximum torque value reached are due to the different values assigned to the nut factor and friction coefficients (K, µ_G_, µ_K_).

The last column indicates the variation of the maximum tightening torque expressed in percentage of the different standards taking into account the value obtained with VDI 2230 Standard [37] as reference. All the values recommended by standards are between 11% and 15% lower than the theoretical reference VDI 2230 values. These deviations can be attributed to the difficulty of the friction coefficient determination as it was found by Bickford [41]. The results of this study show that the coefficient of friction is very difficult to control and predict, since the friction is dependent on many variables.

Christos et al. [27], provided similar results to those obtained by VDI where an uncertainty value is considered depending on the kind of bolted joint: ±35% for unlubricated bolts; ±30% for cad-plated bolts and ±25% for lubricated bolts.

The results obtained in last column of Table 1 show a torque reduction recommendation of 15% (VW) and 11(%) (ISO) for bolted joints.

## 3. Results

### 3.1. Maximum Torque Test

The results obtained in the maximum torque test (until breakage) are indicated in Table 2.

**Figure 3:**
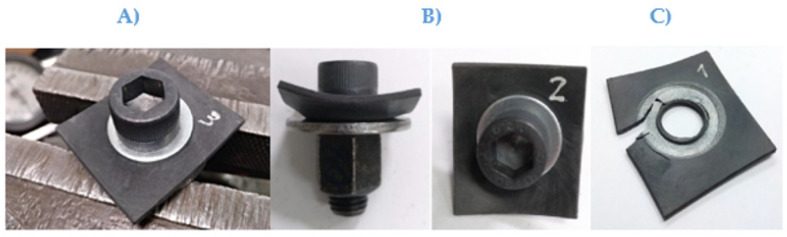
PA66 FG30 (**A**) Prior to the test, (**B**) Deformation at 60 Nm and breakage at 80 Nm. (**C**) Crack at 60 Nm.

- Torque test specimen assembly type 1, PA66-FG30 specimens with no washer:

The maximum tightening torques applied were 43 Nm, 39 Nm, and 45 Nm (Table 2). Failure occurred due to shear breakage, by initially puncturing the perimeter area of the bolt head, followed by a crack appearing from the hole towards one of its sides as it can be observed in Figure 3.

b. Torque test specimen assembly type 2, PA66-FG30 specimens with a washer:

The maximum tightening torques applied were 62 Nm, 64 Nm, and 80 Nm (Table 2). Specimens did not crack until a torque of 80 Nm was reached. As deformation began at 60 Nm, it was estimated that the maximum allowable torque with a washer would be 60 Nm (around 40% higher than without a washer in the worst-case sample).

The results obtained for the washer configuration showed values between 62 and 80 MPa. The difference in values could have been caused by the differences in the assembly configurations. In Figure 3B, a large deformation can be observed without cracking, reaching a maximum torque of 80 Nm. At this point, the test was stopped. However, deformation started at values between 58 and 60 Nm and was visible.

**Figure 4:**
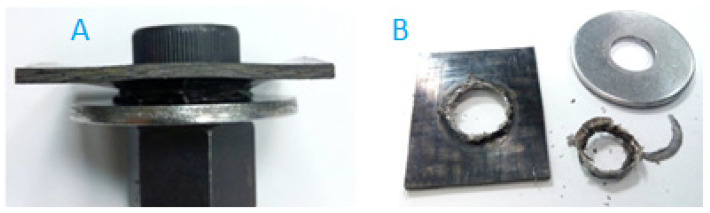
Specimen PA6 FG45 with no washer: (**A**) Ripple failure (M_A_ max = 120 Nm). (**B**) Total collapse (M_A_ = 140 Nm).

In other samples of the type 2 specimen assembly, the breakage appeared immediately when maximum torque was applied without causing large deformation. Figure 3B shows the cracking that took place at a torque of 62 Nm. The cracking started from the area with the highest stress concentration, at the periphery of the washer circumference and spread to one of the sides due to compression failure of the composite.

c. Torque test specimen assembly type 3, PA6-FG45 specimens with no washer:

Maximum torques of 72 Nm, 78 Nm, and 80 Nm were reached. Above these values, the composite plate experienced deformation, resulting in a paraboloid shape (Figure 4A). The failure occurred due to stress concentration under the bolt head. One of the specimens was tested until 140 Nm to intensify and highlight the failure type. Figure 4B depicts how part of the composite was sheared due to the bolt head acting on it, and a circular ring was detached with a similar outer diameter to that of the bolt head.

d. Torque test specimen assembly type 4, PA6-FG45 specimens with a washer:

The maximum torque values in the samples increased by a maximum of 30% and a minimum of 25% before reaching the maximum torques of 90 Nm, 100 Nm, 102 Nm, and 110 Nm.

**Figure 5:**
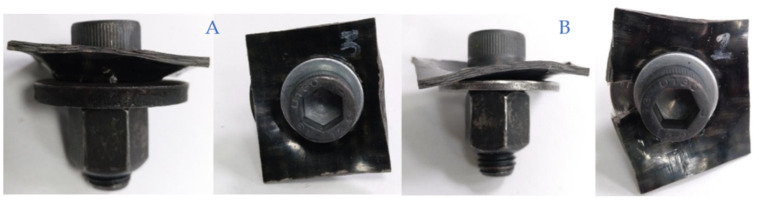
Specimen PA6 FG45 with washer: (**A**) Absence of crack failure after applying the maximum torque. (**B**) Crack failure (Torque > maximum tightening torque).

**Figure 6:**
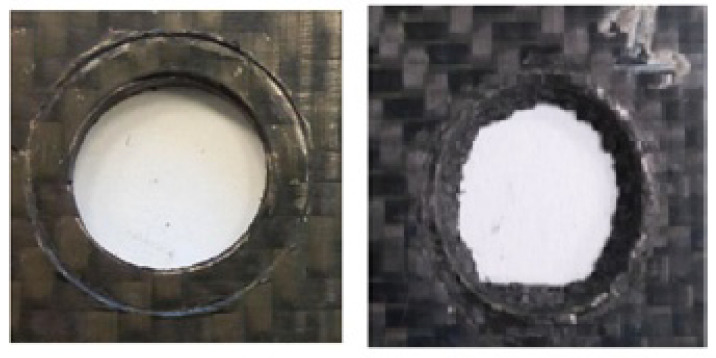
PA66-CF45 plate disassembled at a maximum: (**Left**) Produced by maximum tightening torque. (**Right**) Produced when applied torque is greater than maximum tightening torque.

**Figure 7:**
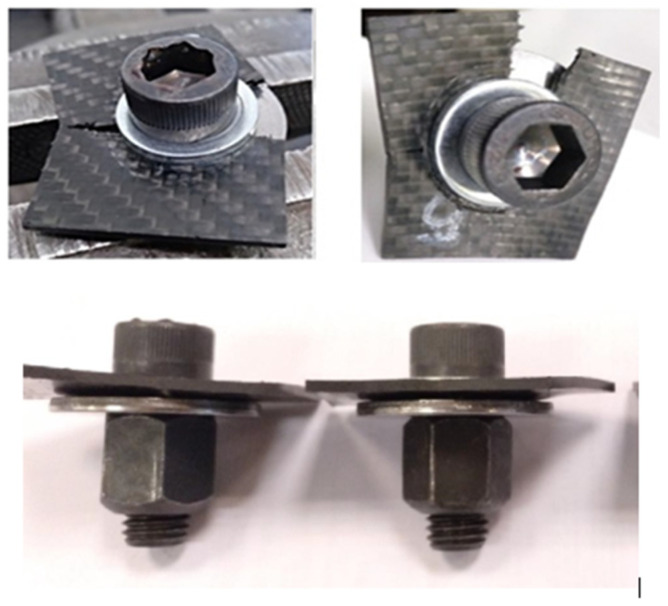
Specimens PA66-CF45 after failure. (**Left**) Crack to 124 Nm without deformation. (**Right**) Fracture to 146 Nm with minimal deformation.

The failure observed in the PA66-FG30 specimens (large deformation or crack) was also found in these specimens. However, with PA6-FG45, the total rupture (crack) occurred after a large deformation of the specimen. This behaviour allowed large deformations without the appearance of breakage. Figure 5A shows a deformed specimen without cracking. Increasing the torque led to failure due to cracking and shear failure did not occur (Figure 5B).

e. Torque test specimen assembly type 5, PA66-CF45 specimens with no washer:

Maximum torque values obtained were 110 Nm and 112 Nm, and 120 Nm. Tests were stopped when the composite plate began to crack, and no plate deformation was observed. Figure 6 (left) shows the bolt head beginning to impinge on the composite plate. Head pull-through failure mode was not obtained however, a visible circumference was observed. Figure 6 (right), illustrates head pull-through failure mode when tightening torque is above 120 Nm.

f. Torque test specimen assembly type 6, PA66-CF45 specimens with a washer:

Maximum torque values of 124 Nm, 144 Nm, and 146 Nm were reached. Three specimens failed due to two cracks: the largest crack grew from the centre towards one of the specimen sides and the smaller at 180° from the main crack as can be observed in Figure 7 (left). Unlike tests with fibreglass specimens, no large deformations were observed (Figure 7 bottom). In one specimen, failure took place with the appearance of the main crack, followed by a secondary crack at 90° which split the composite plate as can be observed in Figure 7 (right).

g. Torque test specimen assembly type 7, C45 Steel Specimens:

Maximum torque of 150 Nm was reached without failure modes. However, in the specimens tested with no washer, the steel specimen was marked, which also happened with the carbon fibre material. The value obtained was used as a reference to compare the values obtained in CFRTP specimen assemblies.

Comparing these values with the maximum torque test carried out, the following conclusions can be obtained: Polyamide PA 66 FG30 (random short fibres) specimens showed a breakage when very low torques are applied (between 39 and 45 Nm with no washer, and between 62 and 80 Nm with a washer). It was concluded that their use was not feasible for the range of the studied products, and therefore, no further tests were carried out.

The PA6-FG45 composite specimens with no washer failed at low and insufficient torques (between 72 and 80 Nm). With a washer, the maximum values reached are higher than the torque required by the VW 01126 [35] for an M10 Class 12.9 (65 Nm) bolted joint, but lower than that required for M12 Class 12.9 (110 Nm). ISO recommends 69 Nm for M10 Class 10.9 and 83 Nm for M10 Class 12.9.

Conclusions: 

CFRTP with continuous carbon-glass and steel support higher torque than automakers’ requirements and ISO standards in the use of bolted and threaded joints. 

The CFRTP with continuous fibreglass supports higher torques than M10 Class 10.9 and slightly higher with M10 Class 12.9. 

The random fibreglass composite (PA66 FG30) does not support enough torques, even with the use of a washer, so it was rejected for subsequent tests.

### 3.2. Loss of Torque Test

The maximum tightening torques were applied to the threaded joints of the composites (82 Nm to PA6-FG45 and 110 Nm to PA66-CF45) and to the steel of the anchor plate. Assemblies were stored in a controlled atmosphere at an ambient temperature of 23 ± 2°C.

The first batch of specimens was disassembled after 7 days and short-term tightness loss was measured. The next batch of specimens was disassembled after 3 years and 3 months and long-term tightness loss was measured. The obtained results are shown in Table 3.

According to the results obtained, the long-term loss of torque for CFRTP components show an average value of 32%. The loss of torque reached a maximum value of 30% for PA6-FG45 (it dropped to 56 Nm from 82 Nm) whereas the maximum loss of torque for PA 6.6-CF (it dropped to 70 Nm from 110 Nm) reached a maximum value of 36%. For steel joints, the loss of torque reached a value of 7%.

The final torques of PA66-CF45 were higher than the maximum recommended torque for bolted and threaded joints with M10 class 10.9 (55 Nm for VW and 69 Nm for ISO standards). 

### 3.3. Case Study: CFRTP Prototype Test

**Figure 8:**
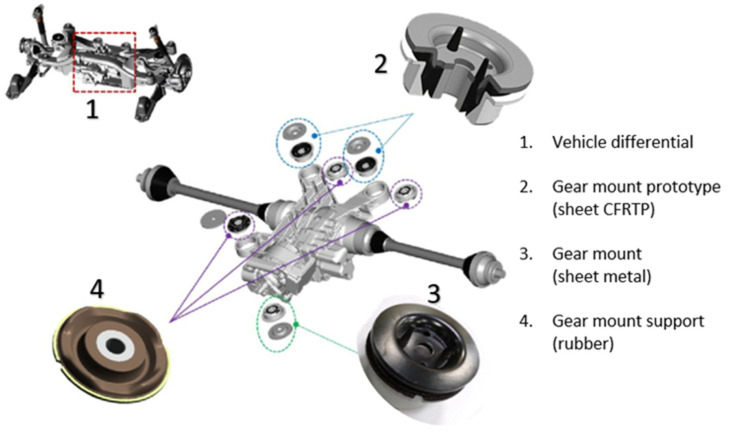
Vehicle Differential System.

To confirm the behaviour of the bolted joints within the range of vehicle antivibration products and shock absorbers, a prototype (Figure 8(2)) of a commercial gear mount (Figure 8(3)) a component of a Differential System (Figure 8) was manufactured by replacing the steel core with CFRTP. 

**Figure 9:**
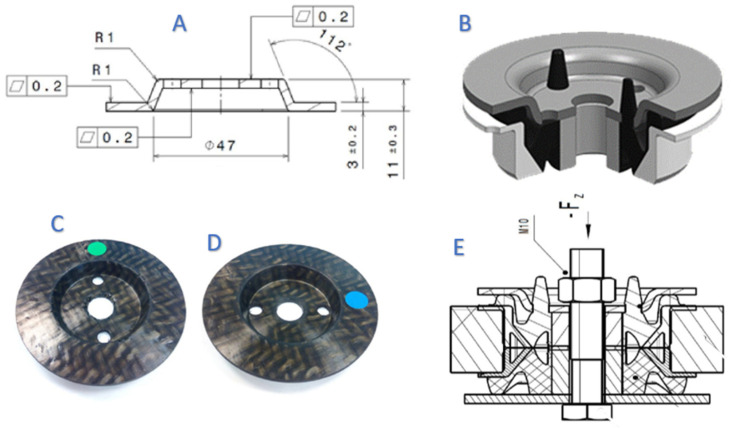
Gear mount prototype. 2D Drawing (**A**). 3D Design (**B**). Specimen CFRTP 3 mm (**C**). Specimen PAF6 FG45 (2.5 mm) (**D**). Assembly (**E**).

The CFRTP part (Figure 9) has the same geometry and dimensions as the steel part, except:

- The radius r1, which was reduced to 1 mm to avoid interlaminar problems.
- The 90° angle, which was extended to 120° to improve the shaping and demoulding.

These differences do not alter the function of the gear mount or require modifications to the vehicle’s differential.

Further, the prototype was sequentially subjected to dynamic and static tests, under conditions recommended by ISO 13003 [42]:

- The type of material tested was a PA6 FG45 (with and without adhesive rubber). The material is a polyamide 6 with Continuous Fibre-Roving Glass-Reinforced Thermoplastic Matrix Polymer (CFRTP, with 45% of bidirectional fibres in a 45° orientation, supplied in laminate plates of 210 × 297 × 0.5 mm, thermoconformed at 240 °C, and manufactured for Bond-laminates, trademark Tepex© Dynalite 102-FG92).
- The method to obtain the test specimens was thermo-conformation. Two different thicknesses (2.5 and 3 mm) using the same material and different compression loads were manufactured.
- Details of the testing machine: Schenk 900H25 servo-hydraulic dynamic testing machine (dynamic and static testing). Application load: up to 25 kN axial load. Frequency range: up to 100 Hz. Stroke: up to 100 mm.
- Testing environment (temperature, humidity, etc.): 23 ± 2°C.
- Area of the test specimens subjected to loads: 360 mm^2^ (with washer).
- Static properties defined by the manufacturers and characterized and validated by Tobalina et al. [32]: Tensile Strength = 402 MPa; Tensile modulus = 22.75 GPa.
- Maximum/minimum applied loads: Conditions recommended for car manufacturers in each test.
- Failure criteria: When the stiffness loss value is greater than 20% considering as reference the stiffness value obtained when the initial tightening torque (1st Axial static Test (Pre-FAT) and the 4th Axial static Test (Post-FAT) is applied and taking into account all the traction–compression cycles recommended by the automobile manufacturers (200,000 cycles). The final results are shown in Table 5 and Figure 14.

#### Test Results

Once assembled in the test ring (Figure 9E), the four required tests were carried out: First the Axial Static Force, Second the Axial Dynamic Force, Third the Fatigue Dynamic Force and fourth Axial Static Force.

**Figure 10:**
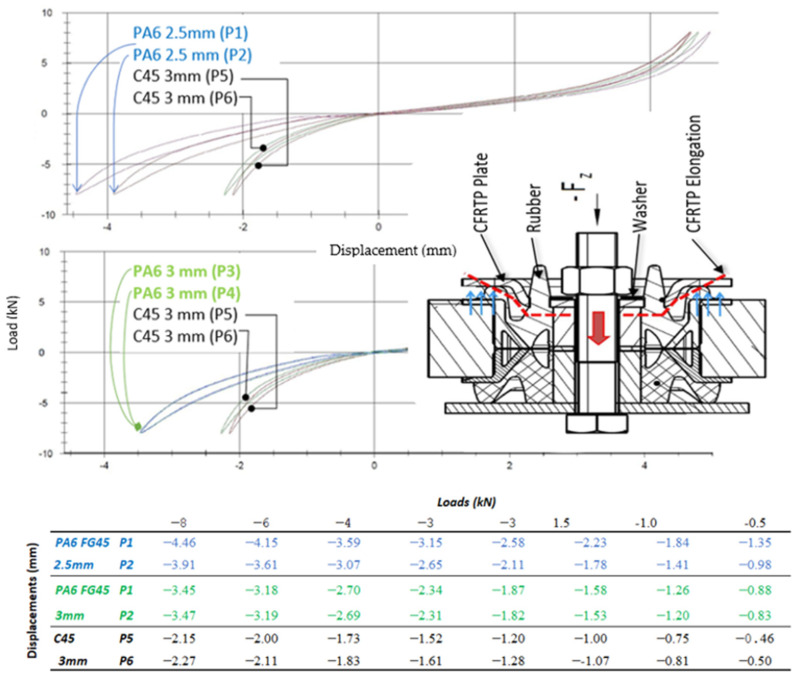
Diagrams of the Axial Static Test (Pre-FAT) of CFRTP and Steel plate deformation caused by Fz force.

Test results were carried out with samples of CFRTP (Figure 10, Green colour), with Steel of thickness 3 mm (Figure 10, Black colour) and with samples with a thickness of 2.5 mm (Figure 10, Blue colour). All the samples were obtained with the same amount of material based on a pre-shaped sheet, but with a variation of the forming pressure:

- Specimen P1: PA6 FG45 with thickness 3 mm.
- Specimen P2: PA6 CF45 with thickness 3 mm.
- Specimen P3: PA FG45 with thickness 2.5 mm.
- Specimen P4: P6 FG45 with thickness 2.5 mm
- Specimens P5 and P6: C45 steel with thickness 3 mm.
- Specimens P1, P2, P3 and P4: 32 g; specimens P5 and P6: 128 g.

a. Axial Static Test results (Pre-FAT):

The vehicle’s assembly tightening torque was applied to the whole piece with the composite plate (Figure 10) and an axial test was subsequently carried out to determine the piece’s stiffness. Specific Conditions Test:

- Three conditioning cycles are carried out to avoid the Mullins effect of vulcanized rubber.
- The maximum force will be 8 kN because, with higher loads, a plastic deformation greater than 0.1 mm occurs.
- Maximum/minimum applied loads: Application of a tensile/compression load of ±8 kN applied to 10 mm/min.
- The test is carried out on the six specimens.

The axial load was applied using common test parameters for such pieces and recording the notation of the produced forces and deformations.

The new Figure 10 (above) shows the CFRTP and steel plate deformation diagrams caused by the tensile/compressive forces (F_z_) in the six samples (Bottom). It only shows negative deformations because they are the most representative forces to ascertain the prototype stiffness when applying the tightening torque. These results show that specimens using PA6 FG45 material compared to steel configurations (parts with the same volume and geometry) produced deformations over the permissible limits:

- Specimens with a thickness of 2.5 mm (average P1 and P2) showed a large dispersion (14%) in the other 4 specimens (6%), therefore, the mean value of its stiffness is not very representative.
- Specimens with 2.5 mm thickness show greater displacements than specimens with a thickness of 3 mm (about 121% in order to CFRTP and 190% comparing with steel).
- All CFRTP plates show too much deformation compared to steel (151% per 3 mm thickness).

These results indicated the following:

- The stiffness of the CFRTP plate increases when the thickness is reduced, while maintaining the same mass. This could be due to a higher formation pressure, which alters the order of the fibres and migrates the matrix to the outside.
- Any CFRTP sample was not permanently broken, cracked, or distorted during testing.
- Despite the high deformation of CFRTP sheets, these facts encouraged us to redesign the part in order to increase its stiffness (deformation is inversely proportional to the stiffness) so that the CFRTPs can be successfully implemented by car manufacturers in structural application.

b. Axial Dynamic Test results (Pre-FAT):

Tobalina et al. [8] demonstrated that CFRTP materials have better vibration absorption and damping properties than steels, which means greater comfort, avoiding vibrations and noise when used in structural elements, with this test it is checked whether the piece vulcanized rubber of the "gear mount" hides these properties. Specific Conditions Test:

- Maximum/minimum applied loads: Application of a tensile/compression load of ±2 kN with a frequency of 25 Hz and an amplitude of ±1 mm.
- Acceptance requirements: It is allowed a tolerance of −20% with respect to the result obtained for a steel plate specimen.
- The test is carried out on the 6 specimens.

Table 4 indicates the average values of the test conditions for two specimens for each type of material). The first column represents the pre-load value applied from 0 to ± 2 kN (dynamic traction/compression). Columns 2, 4, and 8 show the dynamic stiffness values of the steel C45 (3 mm thick), PA6 F45 (2.5 mm thick and 3 mm thick, respectively).

In columns 3, 5, and 7, the values of the damping angle are shown for the steel, the 2.5 mm and 3 mm CFRTP, respectively. The deviations of the stiffness values with respect to the steel part are specified in columns 5 and 9, while columns 7 and 11 represent the deviations of the damping angle with respect to the steel values.

**Figure 11:**
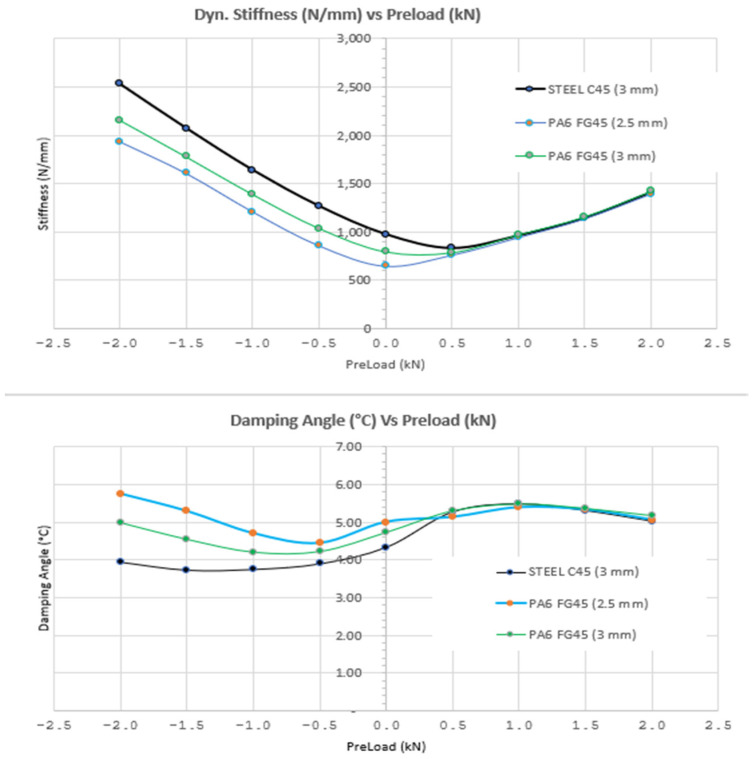
Diagrams of the Axial Dynamic Test (Pre-FAT). Stiffness (top) and damping (bottom) of the CFRTP and steel prior to the Fatigue Test.

These values are plotted in two separated graphs shown in Figure 11 (top): Dynamic Stiffness/Pre-Load. Figure 11 (bottom) Dynamic Angle/Pre-Load.

The following conclusions were found from the obtained results in Table 4:

- CFRTP plates showed lower stiffness values compared to steel plates, which translates to lower impact resistance.
- The 2.5 mm CFRTP plate was discarded due to the exceeded deviation limit established at ±20%, by a 6.2% (from −22.4% to −33.8% with compression force). It was discarded to test for fatigue with this thickness
- The 3 mm CFRTP plate did not exceed the ±20% limit (with a maximum value of −8.5%)
- A lower stiffness value equals a lower capacity to bear the structural loads of the vehicle. Lower stiffness corresponds to a greater deformation of the part.
- The CFRTP plates have a greater damping angle than the steel plates (up to +26% for the plates of 3 mm thickness and up to +46% for the 2.5 mm parts).
- A greater damping angle allows the vehicle to be subject to greater dynamic loads, therefore presenting a better damping behaviour.

Figure 11 shows how increasing the compression force (up to –2 kN) the dynamic stiffness and the damping angle increases. This indicates that reinforcing the CFRTP plate to increase the part stiffness, the damping angle is still greater compared to the steel part, meaning a higher damping capacity, increasing the vehicle comfort.

In conclusion, modifying the geometry and reinforcing the part can lead to an improvement in the structural resistance of the CFRTP plate (higher stiffness), while maintaining the same dynamic behaviour and reducing the weight of the part, which is the main aim of this study.

c. Fatigue test (FAT).

The fatigue test consists of applying cyclical tensile–compression axial load to the gear mount prototype under the following specific conditions:

- Maximum/minimum applied loads: Application of a tensile/compression load of ±5 kN with a frequency of 6 Hz for 200,000 cycles.
- Acceptance requirements: When the stiffness loss value is lower than 20% considering (as reference) the stiffness value obtained when the initial tightening torque (1st Axial static Test (Pre-FAT) and the 4th Axial static Test (Pos-FAT) are applied and taking into account all the traction–compression cycles recommend by the automobile manufacturers (200,000 cycles. The final results are represented and shown in Table 5 and Figure 14.
- The test is carried out on two different prototypes and with two specimens (thickness of 3 mm).

c.1. Testing prototype: Forces and displacements were recorded and controlled to ensure the correct load application allowing to obtain the loss of stiffness and the possible damage suffered by the assembly. After 80,000 cycles, displacements began to increase, and the test was stopped when both the displacement and visual appearance of the sheet degraded up to an unacceptable degree of 98,000 cycles (equivalent to 49% of the defined load cycles).

**Figure 12:**
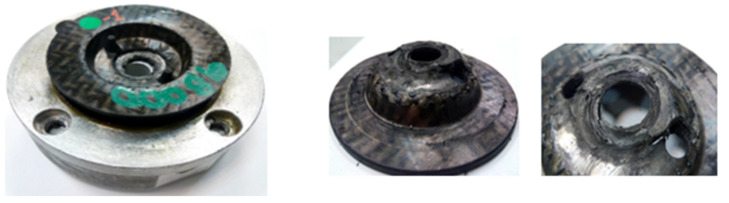
PA6-FG sheet: 3 mm CFRTP deformed after the fatigue test.

The PA6 FG45 specimen can be observed in Figure 12 where the plate of the gear mount is shown after being removed from the fatigue machine. Both the cantilevered upper structure and the transition to the flat zone were not damaged. Plastic deformations nor cracks were observed in these areas. However, shear failure appeared in the torque region. A crack was observed near one of the through-holes, where the rubber stud was inserted to hold the sheet in place during transport. This caused the sheet to tilt because it was not contained by the rubber stud base as the hole was torn.

Figure 12, in the centre, reveals how the fastening area on which the axial fatigue load was applied is plastically deformed, and in such a way that the sheet could not perform its stop function in the assembly, which gave very high displacement values. Figure 10, to the right, depicts the beginning and propagation of the crack in the hole (torque application area). The rest of the composite was not damaged.

**Figure 13:**
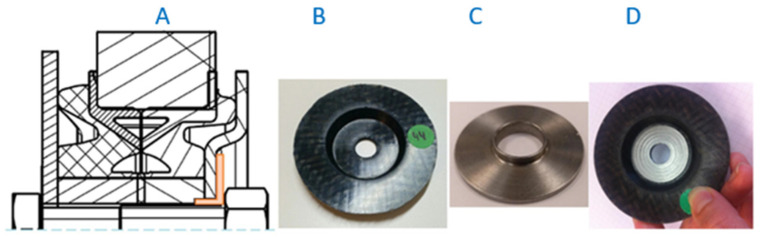
Prototype reinforced: (**A**) Assembly Drawing. (**B**) P6 FG45 sheet. (**C**) Steel insert reinforcement. (**D**) Reinforced sheet after fatigue test (200,000 cycles).

The analysis of the obtained results indicated that the only critical section was the fastening area and the hole layout. This hole was in the perimeter delimiting the zone of the tightening torque. Therefore, it was easy for a high-stress concentration to occur along with very rapid crack growth.

c.2. Alternative solution: the CRTPF plate design is modified. Two holes are eliminated because they are only used for transporting the component. The central hole is enlarged to introduce a steel insert that acts as a fastener during transport and to house the bolt joint to the vehicle. The disadvantage of this solution is that it increases the total weight of the product. The CFRTP board assembly with insert is shown in Figure 13.

**Figure 14:**
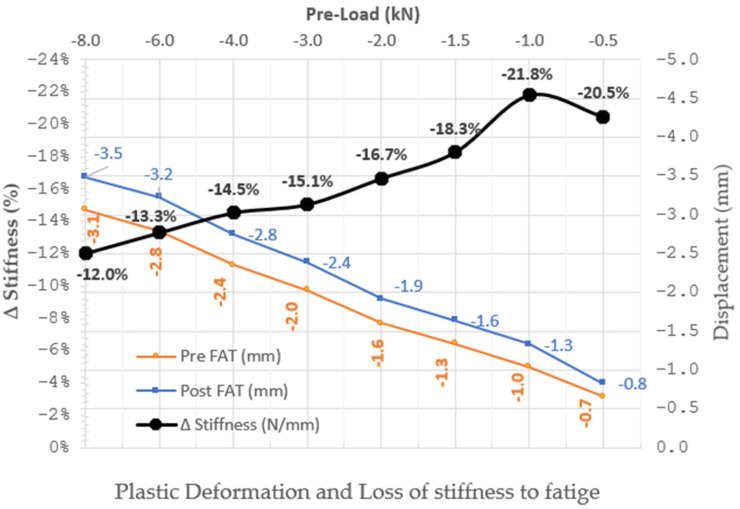
Fatigue Test Results.

In the Modified Prototype (Figure 13), the two plate clamping holes are eliminated, the central hole is enlarged and a steel reinforcement (Figure 13C) is added in order to reinforce the area of application of the tightening torque, also avoiding the need to add a threaded insert (Figure 14) and can remove the washer, partially offsetting the increased weight compared to a board made from 100% CFRTP material.

The new prototype with the hybrid solution was installed on the fatigue machine. In this case, the specimen endured 200,000 cycles without showing visual degradation anywhere. Figure 13D shows the CFRTP specimen after completing 200,000 fatigue test cycles under the initially defined conditions.

d. Axial Static Test (Post-FAT).

The test achieved successfully the first acceptance requirement (exceed 200,000 cycles without apparent deformation). In addition, it must be verified if the specimen has reached the second requirement: loss of stiffness less than 20%.

A second axial static test was carried out to analyse the deformation and stiffness of the new CFRTP specimen before of Fatigue Test (Pre-FAT).

As the sheet structure remained intact, the posterior axial static test was performed to analyse the obtained displacements and stiffness (Post-FAT) and to compare them to those of the previous (pre-FAT) (Table 5 and Figure 14).

From −2 kN (at the highest compression loads), loss of stiffness took place, which met the acceptance criteria; that is, the increase in displacements (Post-FAT) was below 20% versus the values obtained in the previous static axial test (Pre-FAT), except when starting tests (lower compression loads than −1 kN) when the percentage was slightly higher, but the absolute displacement was less than half.

Therefore, the results met the failure criteria set by Standard ISO 13003 [42]. The new prototype design can be considered suitable for replacing steel within the range of the anti-vibration and shock absorber products in automobiles.

## 4. Discussion of the Results and Alternative Solutions

The defined methodology shows the repeatability of the results and it is consistent. Hence it can be considered valid to characterise and analyse the behaviour of bolted joints within the studied range of automotive components.

The strong influence of adding a washer in the torque zone is demonstrated. After implementing this improvement, the maximum torque increased to over 30%. The composite materials studied with a continuous glass (PA6-FG45) or carbon (PA66-CF45), matrix, and both with a built-in washer, provided torques values of up to 90 Nm for fibreglass, and up to 140 Nm for fibreglass carbon fibre. These values fall in line with those recommended by reference standards. The material failure leads to either permanent deformation or cracks that appeared in the specimen.

Over time, the studied composites lost around 32% of the applied maximum torque (more than 3 years), even when they were not subjected to any cyclical or constant load or were subjected to adverse climate cycles. The reason is attributed to the viscoelastic behaviour of thermoplastic materials. These results are not acceptable in the automotive world.

CFRTP materials are 80% lighter than steel and have superior specific mechanical characteristics, which would allow them to replace steel. However, for given geometries and according to certain requirements, the use of CFRTP implies redesigning the part, which means that the direct geometric conversion of steel into CFRTP cannot be carried out.

Adding a washer in the torque zone increases the maximum torque above 30%. CFRTPs with continuous Fibreglass (PA6-FV) or Carbon Glass (PA6.6-FC45), with a washer able to admit torques up to 90 Nm and 140 Nm respectively.

A full-scale gear mount prototype was subjected to a traction–compression fatigue test. As the gear mount core was made of 3 mm-thick steel, the feasibility of CFRTPs was analysed under the same conditions as steel (equal thickness and volume). During the fatigue test, the study case of a prototype made of PA6-FG45 was not capable of withstanding the fatigue test conditions.

At 80,000 cycles, the plate CFRTP of the prototype began to work in the plasticity zone, causing a progressive decrease in stiffness. The failure occurred in the area of contact with the bolted joint. The adopted alternative solution consisted of introducing a metal insert into the anchoring hole to reinforce the bolted joint area in the composite. By repeating the test with the modified design, the CFRTP material sheet withstood cyclical loads during 200,000 cycles with no loss of functionality.

The steel plate part of the gear mount weighed 128 g of the 201 g that the assembly weighed. The composite design weighed 32 g and the reinforcing metal insert provides an additional 15 g of weight, which represented a reduction of 63%. With this solution, the need for the washer and the threaded insert was avoided.

Other possible options whose verifications are beyond the scope of this research study are the following:

- Leave a space in the composite plate for the insert holding element: the insert is inserted into the plate and covered with over-injected composite. In this way, the reinforced joint would support the entire tightening torque load and would guarantee the support of high compression loads and torque without failure, which would be maintained over time.
- Bond the metal insert for the composite structure using an adhesive.

## 5. Conclusions

In this study bolted and threaded joints between continuous fibre-reinforced thermoplastic composites (CFRTP) and metal components are analysed by means of experimental tests. For this purpose, maximum torque that can be applied depending on the material considered and the loss of torque over time was calculated based on experimental tests. After that, the behaviour of the thermoformed CFRTP component with mechanical joints was subsequently analysed in a prototype by means of a fatigue test. 

According to the results obtained, the maximum torque achieved without breakage was at the limit of that required by the automotive industry when the employed material is CFRTP PA66-CF45. For CFRTP PA6-FG45, the maximum torque was below the requirements for this application type. Loss of torque occurred in all CFRTP materials over time, which is simply not acceptable.

All PA66-CF Supports bolted joints up to M12 class 12.9 (Maximum dry torque 110 Nm) and PA6-FG up to M10 Class 12.9 (Maximum dry torque 65 Nm). The values obtained are higher than the torque required by automobile manufacturers, but the tightening torque obtained by the CFRTP with fibreglass, to be accepted, have to guarantee that throughout their useful life they maintain their initial stiffness above 80%. 

This study showed that the anchoring of antivibration and shock absorbing components made with a composite core, and with a thermoplastic matrix reinforced with continuous glass or carbon fibre, cannot be carried out directly on such materials by means of the same type of bolted joints used in current components.

The test on a gear mount prototype with the CFRTP plate and a metal insert in the anchor hole gave a positive result in the successful completion of the fatigue test.

Integrating mechanical elements into the joint areas can solve the problems that appear when using CFRTP in the fastening elements (maximum torque and loss of torque).

The steel plate weight is 64% of the total assembly weight. Using a CFRTP plate with a washer at the joint would reduce the total assembly weight by 63% compared to the current weight. The inclusion of other inserts, such as those defined in the proposed alternatives, would lower this percentage, but it would remain above 50% weight reduction.

Future research should address the design of prototypes with a CFRTP plate and embedded inserts to be used as fasteners covered with over-injection plastic.

## Figures and Tables

**Table 1:** Factor values F_M_ y M_A_ for M12 and M10 steel Allen bolts.

Method		Metric	Class	K	µ _(1)_	F_M_ [kN]	M_A_ [Nm]	% Variation _(2)_
VDI 2230	Equations (3)–(5)	M12 × 1.75	12.9	0.2	0.15	78	185	0%
		M12 × 1.75	12.9		0.09	82	127	0%
	VW 01126-1 (maximum)	M12 × 1.75	12.9		0.09	82	127	0%
Equations (1) and (2)		M12 × 1.75	12.9	0.2		82	197	6.5%
VW 01126-1 (recommendation)		M12 × 1.75	12.9	0.2	0.09	82	110	−15%
		M10 × 1.5	12.9	0.2	0.15	52	65	
		M10 ×1.5	10.9	0.2	0.15	44	55	
ISO Metric (maximum)		M12 ×1.75	12.9	0.2	0.15	72	160	−15%
ISO Metric (recommendation)		M12 × 1.75	12.9	0.2	0.15	61	144	−11%
		M10 × 1.5	12.9	0.2	0.15	44	83	
		M10 × 1.5	10.9	0.2	0.15	37	69	

_(1)_ Lubricated Threads µ = 0.15. Dry Threads µ = 0.09. _(2)_ % Variation = ((MA–MA VDI/MA).

**Table 2:** Maximum torque results (for M12 class 12.9).

Materials	Washer	Maximum Torque [Nm]	Minimum Increase [%]
PA66-FG30	NO	39, 43, 43, 45	
	YES	62, 64, 80, 80	33%
PA6-FG45	NO	72, 78, 78, 80	-
	YES	90, 100, 102, 120	25%
PA66-CF45	NO	110, 112, 112, 120	-
	YES	124, 124, 144, 146	11%
C45 STEEL	NO	≥ 150	-
	YES	≥ 150	-

**Table 3:** Loss of torque results.

Material	Initial Torque [Nm]	Torque [Nm] 13.05.17	Loss [%] 20.15.17	Torque [Nm] 06.08.20	Loss [%] 06.08.20
STEEL C45	110	106	3.6%	-	-
	110	106	3.6%	-	-
	110	-	-	102	7.27%
	110	-	-	104	6.45%
PA66-CF45	110	92	16.3%	-	-
	110	94	14.5%	-	-
	110	-	-	73	33.6%
	110	-	-	70	36.36%
PA6-FG45	82	72	12.2%	-	-
	82	69	15.8%	-	-
	82	-	-	58	28.4%
	82	-	-	56	30%

**Table 4:** Results from the Axial dynamic Test (Pre-FAT) of the assembly prototype.

Dynamic Load: ±2 kN. Frequency: 25 Hz. Amplitude: ±1 mm										
	STEEL C45 (3 mm)		PA6 FG45. (2.5 mm)				PA6 FG45 (3 mm)			
Loads	Stiffness	Angle	Stiffness		Angle		Stiffness		Angle	
kN	(N/mm)	(°C)	(N/mm)	Desv (%)	(°C)	Desv (%)	(N/mm)	Desv (%)	(°C)	Desv (%)
−2.0	2533	3.9	1936	−23.6%	5.7	46.1%	2159	−14.8%	5.0	26.00%
−1.5	2074	3.7	1608	−22.4%	5.3	41.9%	1780	−14.2%	4.5	21.7%
−1.0	1641	3.7	1211	−26.2%	4.0	25.3%	1391	−15.3%	4.2	12.0%
−0.5	1268	3.9	859	−32.2%	4.4	14.0%	1039	−18.0%	4.2	8.2%
0.0	976	4.9	647	−33.8%	5.0	15.4%	796	−18.5%	4.7	9.0%
0.5	830	5.3	761	−8.3%	5.1	−2.8%	787	−5.3%	5.3	−0.4%
1.0	959	5.5	943	−1.6%	5.4	1.9%	971	1.3%	5.5	0.0%
1.5	1141	5.3	1139	−0.2%	5.2	−0.3%	1158	1.5%	5.4	0.9%
2.0	1407	3.0	1395	−0.9%	5.1	0.6%	1422	1.0%	5.2	2.8%

**Table 5:** Fatigue (200,000 cycles). Results with Reinforced specimen CFRTP Thickness 3 mm.

Static Axial Test Results							
Loads [kN]	−8	−6	−4	−3	−2	−1	−0.50
Displacements. Pre–FAT [mm]	−3.07	−2.8	−2.35	−2.03	−1.6	−1.04	−0.66
Displacements. Post–FAT [mm]	−3.49	−3.23	−2.75	−2.39	−1.92	−1.33	−0.83
Δ displacement [%]	13.68	15.36	17.02	17.73	20.00	27.88	25.76
Stiffness Pre–FAT [N/mm]	2606	2143	1702	1478	1250	1119	961
Stiffness Post–FAT	2292	1858	1455	1255	1042	752	602
Δ Stiffness [%]	−12.03	−13.31	−14.55	−15.06	−16.67	−21.80	−20.48

## Data Availability

The data presented in this study are available on request from the corresponding author.

## Abbreviations

**Nomenclature**	**Name**
CF	Carbon-Fibre or Carbon-Fiber.
CFRTP	Continuous Fibre-Reinforced Thermoplastic Composites.
FRP	Fibre-Reinforced Polymers.
C45	High quality, high strength 0.45% carbon steel EN 1.083/2, ASTM 1045).
FG	FibreGlass or FiberGlass.
FRTP	Fibre-Reinforced Thermoplastic Polymers.
GRP	Glass-Reinforced Plastic. = GFRP: Glass-Fibre-Reinforced Plastic.
PA6	Thermoplastic Polyamide produced by the polymerization of caprolactam.
PA66 FG30	Random short FibreGlass 30%-reinforced Polyamide.
PA6 FG45	Continuous FibreGlass 45%-reinforced Polyamide.
PA66	Thermoplastic Polyamide obtained by polycondensation of adipic acid with hexamethylenediamine. It is a thermoplastic composite.
PA66 CF45	Continuous Carbon-Fibre 45% reinforced Polyamide.
PBT	Polybutylene Terephthalate. It is a thermoplastic polymer-type polyester.

## References

[1] Mayyas A., Qattawi A., Omar M., Shan D. (2012). Design for sustainability in automotive industry: A comprehensive review. Renew. Sustain. Energy Rev..

[2] Kuram E., Ozcelik B., Yilmaz F. (2016). The influence of recycling number on the mechanical, chemical, thermal and rheological properties of poly(butylene terephthalate)/polycarbonate binary blend and glass-fibre-reinforced composite. J. Thermoplast. Compos. Mater..

[3] Reis J.P., de Moura M., Samborski S. (2020). Thermoplastic composites and their promising applications in joining and repair composites structures: A review. Materials.

[4] Şimşek B., Uygunoğlu T. (2018). A full factorial-based desirability function approach to investigate optimal mixture ratio of polymer concrete. Polym. Compos..

[5] Roenner N., Yuan H., Krämer R.H., Rein G. (2019). Computational study of how inert additives affect the flammability of a polymer. Fire Saf. J..

[6] Yang W., Hu Y., Tai Q., Lu H., Song L., Yuen R.K.K. (2011). Fire and mechanical performance of nanoclay reinforced glass-fiber/PBT composites containing aluminum hypophosphite particles. Compos. Part A Appl. Sci. Manuf..

[7] Zhang D., He M., Qin S., Yu J., Guo J., Xu G. (2018). Study on dynamic mechanical, thermal, and mechanical properties of long glass fiber reinforced thermoplastic polyurethane/poly(butylene terephthalate) composites. Polym. Compos..

[8] Tobalina-Baldeon D., Sanz-Adan F., Martinez-Calvo M.A., Santamaría-Pena J. (2020). Dynamic tensile stress-compressive stress behavior of thermoplastic matrix composite materials reinforced with continuous fiber for automotive damping and anti-vibration structural elements. Materials.

[9] Williams G., Trask R., Bond I. (2007). A self-healing carbon fibre reinforced polymer for aerospace applications. Compos. Part A Appl. Sci. Manuf..

[10] Pramanik A., Basak A.K., Dong Y., Sarker P.K., Uddin M.S., Littlefair G., Dixit A.R., Chattopadhyaya S. (2017). Joining of carbon fibre reinforced polymer (CFRP) composites and aluminium alloys—A review. Compos. Part A Appl. Sci. Manuf..

[11] Volkersen O. (1938). The Rivet-Force Distribution in Tension-Stressed Riveted Joints with Constant Sheet Thicknesess. Luftfahrtforschung.

[12] Hart-Smith L.J. (1985). Bonded-bolted composite joints. J. Aircr..

[13] Ishii K., Imanaka M., Nakayama H., Kodama H. (1998). Fatigue failure criterion of adhesively bonded CFRP/metal joints under multiaxial stress conditions. Compos. Part A Appl. Sci. Manuf..

[14] Tobalina-Baldeón D., Sanz-Adan F. (2019). Characterization of an adhesive bonding between continuous fiber reinforced thermoplastic (CFRTP) composites and vulcanized rubber under a shear load. Dyna.

[15] Roesner A., Olowinsky A., Gillner A. (2013). Long term stability of laser joined plastic metal parts. Phys. Procedia.

[16] Zhang Z., Shan J., Tan X., Zhang J. (2017). Improvement of the laser joining of CFRP and aluminum via laser pre-treatment. Int. J. Adv. Manuf. Technol..

[17] Scarselli G., Quan D., Murphy N., Deegan B., Dowling D., Ivankovic A. (2021). Adhesion Improvement of Thermoplastics-Based Composites by Atmospheric Plasma and UV Treatments. Appl. Compos. Mater..

[18] Bolt S. (2014). Ultrasonic Plastic Welding of Carbon Fiber Reinforced Polyamide 6 to Aluminum and Steel.

[19] Suzuki K., Ohsawa I., Takahashi J., Uzawa K. (2007). Numerical study on ultrasonic welding joint for CFRTP. Technology.

[20] Lambiase F., Genna S., Leone C., Paoletti A. (2017). Laser-assisted direct-joining of carbon fibre reinforced plastic with thermosetting matrix to polycarbonate sheets. Opt. Laser Technol..

[21] Tan X., Zhang J., Shan J., Yang S., Ren J. (2015). Characteristics and formation mechanism of porosities in CFRP during laser joining of CFRP and steel. Compos. Part B Eng..

[22] Jiao J., Wang Q., Wang F., Zan S., Zhang W. (2017). Numerical and experimental investigation on joining CFRTP and stainless steel using fiber lasers. J. Mater. Process. Technol..

[23] Lambiase F., Paoletti A., Grossi V., Genna S. (2017). Improving energy efficiency in friction assisted joining of metals and polymers. J. Mater. Process. Technol..

[24] McCarthy C., McCarthy M., Irving P.E., Soutis D. (2015). Design and failure analysis of composite bolted joints for aerospace composites. Polymer Composites in the Aerospace Industry.

[25] Ramkumar R., Saether E., Cheng D., Vankyya V.B. (1986). Design Guide for Bolted Joints in Composite Structures.

[26] Hart-Smith L.J. (2004). Bolted joint analyses for composite structures-Current empirical methods and future scientific prospects. Joining and Repair of Composite Structures.

[27] Chamis C.C. (1990). Simplified Procedures for Designing Composite Bolted Joints. J. Reinf. Plast. Compos..

[28] ASTM (1990). Test methods assess properties of high modulus fibers, interlaminar properties, laminates, sandwich construction, and structural test methods. USA Annual Book of ASTM Standards ASTM International.

[29] Tsai S.W., Wu E.M. (1971). A General Theory of Strength for Anisotropic Materials. J. Compos. Mater..

[30] Puck A., Schürmann H. (1998). Failure analysis of FRP laminates by means of physically based phenomenological models. Compos. Sci. Technol..

[31] DeTeresa S.J., Freeman D.C., Groves S.E. (2004). The Effects of Through-thickness Compression on the Interlaminar Shear Response of Laminated Fiber Composites. J. Compos. Mater..

[32] Tobalina D., Sanz-Adan F., Lostado-Lorza R., Martínez-Calvo M., Santamaría-Peña J., Sanz-Peña I., Somovilla-Gómez F., Eynard B., Nigrelli V., Oliveri S., Peris-Fajarnes G., Rizzuti S (2017). Characterization of a Composite Material Reinforced with Vulcanized Rubber. Advances on Mechanics, Design, Engineering and Manufacturing.

[33] Gómez C., Mira J., Carrión-Vilches F.J., Cavas F. (2021). Dynamic moduli of polybutylene terephthalate glass fiber reinforced in high-temperature environments. Materials.

[34] Carlsson L.A., Adams D.F., Pipes R.B. (2014). Experimental Characterization of Advanced Composite Materials.

[35] Volkswagen (2017). VW 01126-1. Joining Systems. Tightening Torques for Threaded Joints.

[36] Volkswagen (2018). VW 01126-2. Assembly technique. Tightening Torques for Threaded Assemblies Beyond.

[37] VDI 2230-1 (2018). Systematic Calculation of Highly Stressed Bolted Joints-Joints with One Cylindrical Bolt.

[38] Hosoi A., Kawada H. (2018). Fatigue life prediction for transverse crack initiation of CFRP cross-ply and quasi-isotropic laminates. Materials.

[39] ISO 16047:2005/AMD 1:2012 (2012). Fasteners—Torque/Clamp Force Testing-amendment 1. ISO/TC2/SC11 Fasteners whit Metric Exernal Thread.

[40] ISO 898-1 (2013). Mechanical Properties of Fasteners Made of Carbon Steel and Alloy Steel—Part 1: Bolts, Screws and Studs with Specified Property Classes—Coarse Thread and Fine Pitch Thread. ISO/TC2/SC11 Fasteneres with Metric External Thread.

[41] Bickford J., Faulkner L.L., Non-Gasketed Join (2008). An Introduction to the Design and Behavior of Bolted Joints.

[42] ISO 13003 (2003). Fibre-Reinforced Plastics–Determination of Fatigue Properties under Cyclic Loading Conditions. ISO/TC61/SC13 Compsites and Reinforcement Fibres.

